# Nanozyme-Based Lateral Flow Immunoassay (LFIA) for Extracellular Vesicle Detection

**DOI:** 10.3390/bios12070490

**Published:** 2022-07-06

**Authors:** Baihui Wang, Amanda Moyano, José María Duque, Luis Sánchez, Guillermo García-Santos, Luis J. García Flórez, Esther Serrano-Pertierra, María del Carmen Blanco-López

**Affiliations:** 1 Department of Physical and Analytical Chemistry, Institute of Biotechnology of Asturias, University of Oviedo, c/Julián Clavería 8, 33006 Oviedo, Spain; uo266958@uniovi.es (B.W.); amanda16_3@hotmail.com (A.M.); 2 Hospital Universitario San Agustín, 33401 Avilés, Spain; josemaria.duque@sespa.es (J.M.D.); luis.sanchez@sespa.es (L.S.); 3 Department of Medicine, University of Oviedo, 33006 Oviedo, Spain; 4 Department of General and Digestive Surgery, Hospital Universitario Central de Asturias (HUCA), 33011 Oviedo, Spain; guillermo.garcias@sespa.es (G.G.-S.); garciafluis@uniovi.es (L.J.G.F.); 5 Health Research Institute of the Principality of Asturias (ISPA), 33011 Oviedo, Spain; 6 Department of Surgery, University of Oviedo, 33006 Oviedo, Spain

**Keywords:** extracellular vesicles, iron oxide superparamagnetic nanoparticles (Fe_3_O_4_ SMNPs), lateral flow immunoassay (LFIA), nanozyme

## Abstract

Extracellular vesicles (EVs) are biological nanoparticles of great interest as novel sources of biomarkers and as drug delivery systems for personalized therapies. The research in the field and clinical applications require rapid quantification. In this study, we have developed a novel lateral flow immunoassay (LFIA) system based on Fe_3_O_4_ nanozymes for extracellular vesicle (EV) detection. Iron oxide superparamagnetic nanoparticles (Fe_3_O_4_ MNPs) have been reported as peroxidase-like mimetic systems and competent colorimetric labels. The peroxidase-like capabilities of MNPs coated with fatty acids of different chain lengths (oleic acid, myristic acid, and lauric acid) were evaluated in solution with H_2_O_2_ and 3,3,5,5-tetramethylbenzidine (TMB) as well as on strips by biotin–neutravidin affinity assay. As a result, MNPs coated with oleic acid were applied as colorimetric labels and applied to detect plasma-derived EVs in LFIAs via their nanozyme effects. The visual signals of test lines were significantly enhanced, and the limit of detection (LOD) was reduced from 5.73 × 10^7^ EVs/μL to 2.49 × 10^7^ EVs/μL. Our work demonstrated the potential of these MNPs as reporter labels and as nanozyme probes for the development of a simple tool to detect EVs, which have proven to be useful biomarkers in a wide variety of diseases.

## 1. Introduction

Iron oxide superparamagnetic nanoparticles (Fe_3_O_4_ MNPs) have attracted considerable scientific interest due to their superparamagnetic properties, biocompatibility, and non-toxicity, resulting in a wide range of biomedical and technological applications. For example, Fe_3_O_4_ MNPs have been applied in energy storage [1]; tissue engineering [2]; protein, DNA, and cell separation from samples [3,4]; biosensing [5]; drug-delivery and -targeting [6,7,8]; magnetic resonance imaging (MRI) [9,10,11]; and as mediators of heat for cancer therapy (hyperthermia) [12]. 

The work of Gao et al. [13] first reported that Fe_3_O_4_ nanoparticles have intrinsic peroxidase-like activity, catalyzing the oxidation of chromogenic substrates (TMB, DAB, and OPD) as the natural horseradish peroxidase. The word “nanozyme” has been coined to describe this kind of enzyme-mimicking nanomaterial, since enzymes and nanozymes share similar catalytic properties [14]. These nanomaterials with enzyme-mimetic activity have shown several advantages over natural enzymes. Fe_3_O_4_ MNPs nanozymes are much more cost-effective as well as more suitable for large-scale production; they also exhibit robustness for diverse uses, ease of modification and large surface areas, which allows them to be conjugated to various ligands for multifunctionalities, such as antibodies [15], peptides [16], and small molecules [17]. Considering these strengths, Fe_3_O_4_ nanozymes have shown potential in a wide range of applications, as reviewed in [18,19,20]. Specifically, by merging distinctive physicochemical features and catalytic properties, numerous nanozyme-based platform technologies have been established, for bioanalysis [21], disease diagnosis [22,23], and therapy [24,25]. Noteworthy among these technologies are nanozyme-based biosensors. However, in the case of ferrum-based nanozymes, reports in the literature on their use in paper-based biosensors are still scarce. Duan et al. [26] successfully developed a nanozyme-based immunoassay using Fe_3_O_4_ magnetic NPs for Ebola virus detection on paper strips. Sensitivity was improved 100-fold in comparison with the standard colloidal gold-based LFIA. Thus, the use of iron oxide MNPs as nanozymes has shown great potential in the detection of biomarkers. In this specific field, extracellular vesicles (EVs) have become targets of interest since they are present in bodily fluids and are involved in intercellular communication in physiological and pathological processes [27,28,29]. The generic term EV includes those vesicles with an endosomal origin (exosomes) or formed by membrane budding (microvesicles/microparticles). EV cargos, such as proteins, nucleic acids, and lipids, are considered powerful sources of biomarkers. In addition, EV levels may be altered under pathological conditions, as in chronic fatigue syndrome [30]. Therefore, EVs have become significant targets in diagnostics and have been determined in cancers [31,32] and non-cancerous diseases [33,34,35]. Nevertheless, EVs are a relatively new type of targets for bioanalysis and detection, and most standard analytical methods have insufficient sensitivity and throughput to be usable in EV detection, let alone for clinical use [36,37], as the quantification needs in the field demand rapid and simplified methods of analysis [38]. Active research is thus underway to overcome these challenges. 

Our research group has developed LFIAs for plasma-derived EVs and explored different types of MNPs with the double aim of facilitating the isolation step from biological media and reporter labels in LFIAs [39,40,41]. In this work, we have evaluated the peroxidase-like activity of Fe_3_O_4_ MNPs coated with three different double layers of fatty acids: MNP-OA: oleic acid-CH_3_(CH_2_)_7_CH=CH(CH_2_)_7_COOH; MNP-LA: lauric acid- CH_3_(CH_2_)_10_COOH; and MNP-MA: myristic acid-CH_3_(CH_2_)_12_COOH. The coating of MNPs with fatty acids provides several advantages: their stability in water increases, they are biocompatible for biomedical uses, and the presence of carboxylic groups makes them suitable for subsequent bioconjugation [42,43]. To this end, TMB was employed as the chromogenic substrate to investigate color development both in solution and in lateral flow affinity assays using different concentrations of Fe_3_O_4_ MNPs. In addition, we have developed a nanozyme-mediated signal readout lateral flow immunoassay for detection of plasma-derived EVs to improve the sensitivity of the system. For this purpose, various concentrations of Fe_3_O_4_ MNPs with different coatings were added into TMB and H_2_O_2_ to generate colored substrates and absorbances were measured with a UV–Vis spectrophotometer. For immunoaffinity tests on strips, neutravidin-conjugated Fe_3_O_4_ MNPs left brown test lines on membranes when LFIA was accomplished. The strips were immersed int TMB and H_2_O_2_ mixed solution for signal amplification. A lateral flow optical reader was utilized to measure the color intensity of the test lines before and after peroxide reactions. LFIA paper-based biosensors were further explored for EV detection. Anti-CD9 and anti-IgG were immobilized in the test lines and control lines on the membranes, respectively. Antibody anti-CD63-coupled Fe_3_O_4_ MNPs presented on the test line with a brown color and the completed biosensors were bathed in the substrate mixture for signal enhancement and measured with the reader before and after nanozyme effect.

## 2. Materials and Methods

### 2.1. Chemicals and Reagents

Superparamagnetic magnetite nanoparticles (MNPs) were synthesized by co-precipitation and characterized as reported by Bica et al. [42]. The particles were then coated with biocompatible surfactants: oleic acid (MNP-OA), lauric acid (MNP-LA), and myristic acid (MNP-MA). 

Neutravidin, biotin-BSA, N-(3-Dimethylaminopropyl)-N′-ethyl carbodiimide (EDC), N-Hydroxy succinimide (NHS), bovine serum albumin (BSA), anti-mouse IgG, 3,3′,5,5′-Tetramethylbenzidine (TMB), and hydrogen peroxide (H_2_O_2_) were purchased from Merck (Darmstadt, Germany). Monoclonal antibodies anti-CD63 and anti-CD9 were acquired from Immunostep S.L (Salamanca, Spain). The other reagents used in this study were of analytical grade.

Nitrocellulose membranes (UniSart CN95) were purchased from Sartorius (Madrid, Spain). The other materials used were glass fiber sample pads (GFCP001000, Millipore, Darmstadt, Germany), backing cards (KN-V1080, Kenoshatapes, Amstelveen, The Netherlands) and absorbent pads (Whatman, Piscataway, USA).

Based on previous results, the sample buffer consisted of 10 mM phosphate-buffered saline (PBS), pH 7.4, with 0.5% Tween-20 and 1% BSA.

### 2.2. Equipment

Analysis of absorbance of magnetic nanoparticles with various coatings was achieved with a UV–Vis spectrophotometer (PG Instrument, LTD) together with UV/Win Spectrophotometer Software. An IsoFlow reagent dispensing system (Imagene Technology, Hanover, USA) was used to dispense the detection lines (dispense rate: 0.100 μL/mm) and the strips were cut with a guillotine Fellowes Gamma (Madrid, Spain). A portable strip reader ESE Quant LR3 lateral flow system (Qiagen Inc., Hilden, Germany) was used to quantify the intensity of the test line by means of reflectance measurements.

### 2.3. Functionalization of MNPs

The carboxylic groups were activated using carbodiimide chemistry at a molar ratio EDC/NHS of 1.1. The MNPs were then incubated for 20 min under shaking. Then, the desired concentrations of neutravidin or anti-CD63 antibody were added and incubated for 4 h. The mixture was blocked with 1% BSA and further separated by a magnet for 10 min. Lastly, the conjugates were dispersed in 10 mM PBS, pH 7.4.

### 2.4. Evaluation of Peroxidase-like Activity and Signal Enhancement

To test the catalytic activity of three MNPs in solution, TMB and H_2_O_2_ were added to different concentrations of MNPs. The oxidation of TMB was monitored by measuring the absorbance at 652 nm and 25 °C after 10 min using a UV–Vis spectrometer. The experiments were performed in triplicate, and the calibration curves were elaborated to study the effects of different coatings.

### 2.5. Isolation and Characterization of Plasma-Derived Extracellular Vesicles

Plasma samples were collected after written informed consent was obtained and with the approval of the Ethics Committee of the Hospital Universitario San Agustín (Avilés, Spain). Peripheral venous blood was collected in 10 mL tubes with EDTA as an anticoagulant after discarding the first milliliter and processed within 30 min of collection. Blood was first centrifuged for 30 min at 1550× g to remove cells. Aliquots of plasma were stored at −80 °C until use.

EVs from healthy controls were isolated with ExoQuickTM precipitation solution (System Biosciences, Palo Alto, CA, USA), following the manufacturer’s instructions. EVs were characterized in terms of size and concentration using a NanoSight LM10 instrument (Malvern, Worcestershire, UK) and NTA 3.1 software at Nanovex Biotechnologies S.L (Asturias, Spain). Samples were diluted in 10 mM HEPES 7.4 to achieve a particle concentration ranging from 10^7^ to 10^9^ particles/mL.

### 2.6. Lateral Flow Assays

#### 2.6.1. Preparation of the Lateral Flow Strips

A nitrocellulose membrane was incorporated onto a plastic backing card to give robustness to the membrane. For the affinity tests, a biotin-BSA (1 mg/mL) test line was immobilized on the membrane. For the LFIA tests, antibodies anti-CD9 and anti-IgG at a concentration of 1 mg/mL were dispensed as the test line and control line, respectively. The reagents were dispensed across the membrane at a rate of 0.100 μL/mm. The sample pad and the absorbent pad were then assembled onto the backing card with an overlap of around 2 mm. The complete strip was cut into individual 4 mm strips. 

#### 2.6.2. Lateral Flow Assays

The affinity assays between biotin and neutravidin on paper-based biosensor were performed as a model study for LFIA, since the vitamin biotin and the protein avidin, including its analogue streptavidin and neutravidin, bind together irreversibly [44]. Then, 10 μL of the MNP–neutravidin conjugates were transferred into microtubes with running buffer to a final volume of 100 μL. The strips were then added and allowed to run for 20 min. 

A similar procedure was followed for the detection of EVs. A range of concentrations of EV samples were homogenized with the detection antibody coupled to the MNPs. Then, the strips were added and allowed to run for 15 min.

**Figure 1:**
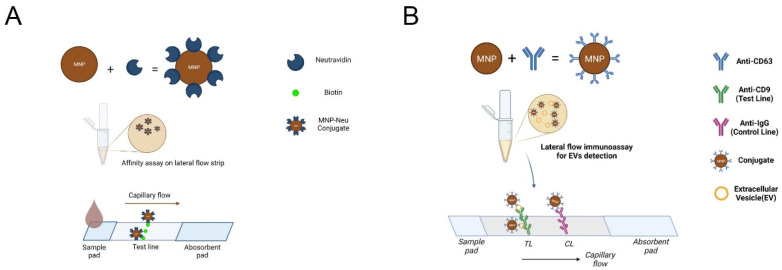
Schematic representation of the (**A**) lateral flow affinity assay (biotin–neutravidin) and the (**B**) lateral flow immunoassay for EV detection, using MNPs as reporter labels. Figure 1 was created using https://biorender.com/.

Schematics for both types of lateral flow assays are shown in Figure 1.

## 3. Results

### 3.1. Nanozyme Activity of MNPs Coated with Fatty Acids of Different Chain Lengths

**Figure 2:**
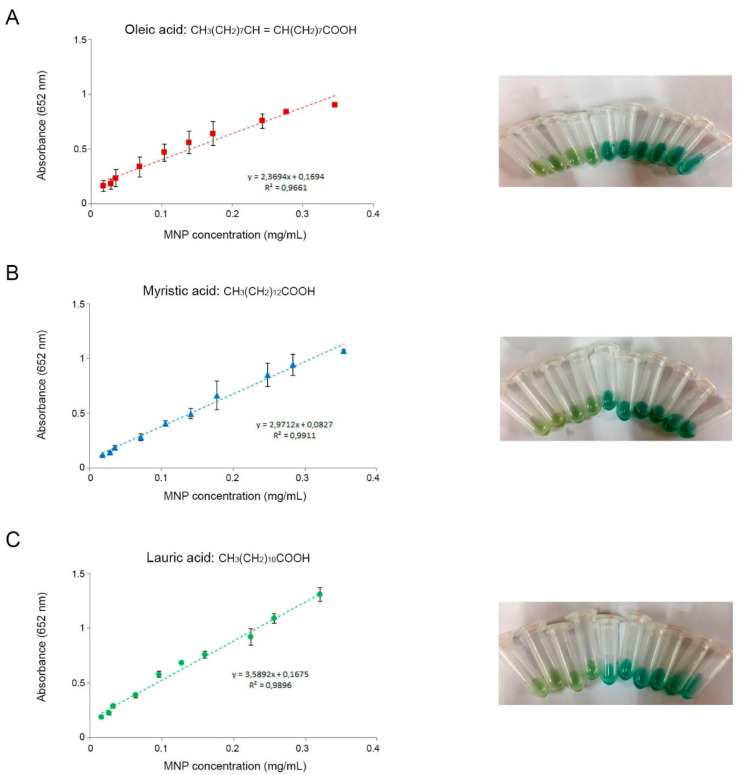
Calibration curves using the nanozyme properties (peroxidase-like activity) of MNPs coated with (**A**) oleic acid, (**B**) myristic acid, and (**C**) lauric acid. Graphs show the mean ± SD of three independent experiments. Representative images of the color developments at the different concentrations tested are shown for each type of MNP.

To evaluate peroxidase-like activity and the potential effect of chain length, the chromogenic substrate TMB was selected. TMB is oxidized faster than other HRP substrates, thus enabling a quick development of color. A range of concentrations of MNP-OA, MNP-MA, and MNP-LA were employed to catalyze TMB in solution when H_2_O_2_ was present. In general, all the MNPs catalyzed the oxidation of TMB, mimicking peroxidase, and turning the colors of the solutions from transparent to varying shades of green (Figure 2). Higher nanozyme concentrations yielded higher absorbance values, thus indicating higher rates of reactions. No significant differences were found regarding the different coatings of the MNPs. Figure 1 shows the calibration curves obtained for the three types of MNPs after measuring the absorbance. A straight line fitted best for the set of data in all cases, with similar coefficients of determination (R^2^). Therefore, all three types of MNPs were further used as reporter labels on paper-based biosensors.

### 3.2. Nanozymes as Labels for Lateral Flow Assays 

**Figure 3:**
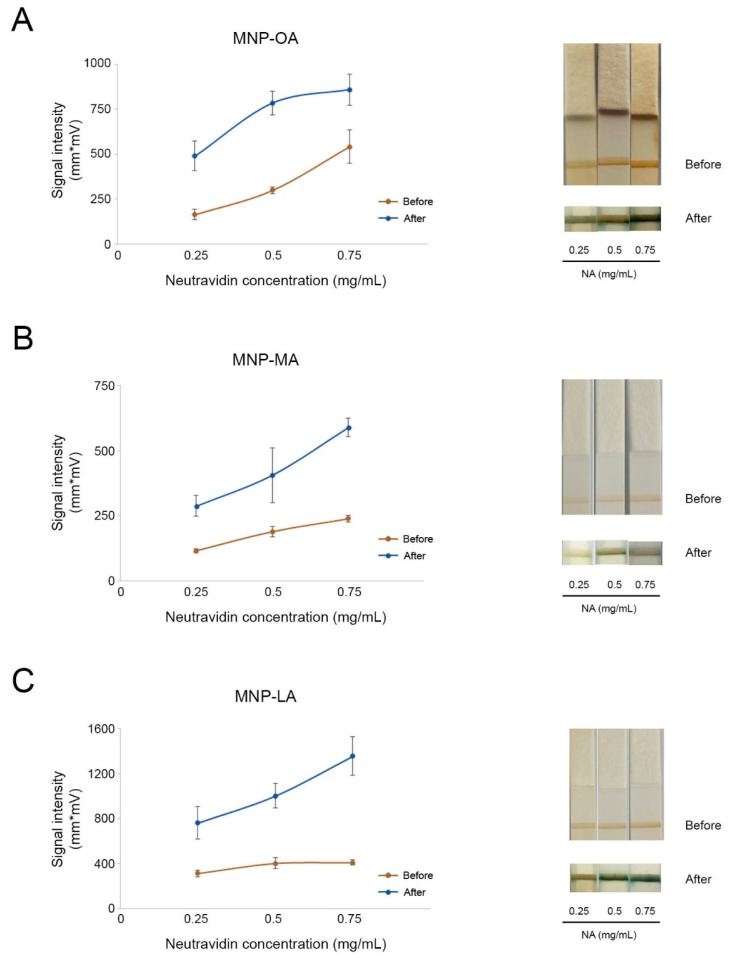
Nanozyme-mediated signal enhancement on lateral flow strips. MNP-OA (**A**), MNP-MA (**B**), and MNP-LA (**C**) were functionalized with different concentrations of neutravidin (0.25 mg/mL, 0.5 mg/mL, 0.75 mg/mL) and used as reporter labels for affinity assays. Signal intensities were measured before (brown lines) and after (blue lines) signal enhancement. Graphs show the means ± SDs of three independent experiments. Representative LFA strips before and after enhancement are shown for each concentration.

Since the three types of particles showed good peroxidase-like properties, their performance in a paper-based sensor was evaluated. As a model study for LFIA, an affinity test between biotin and neutravidin was carried out. To this end, the MNPs were conjugated with three different concentrations of neutravidin (0.25, 0.50, and 0.75 mg/mL). The strips were completely immersed in the substrate solution for 15 min. The intensity of the test lines was measured before and after immersion (Figure 3).

As shown in Figure 3, MNPs exhibited their abilities as optical labels by recognizing biotin and leaving a brown test line on the strip, which can be measured by an optical signal. Nevertheless, MNPs with different coatings performed differently as probes in LFIA. Overall, the intensity of the test lines before treatment with the substrate solution increased when using higher concentrations of conjugated neutravidin. This trend was more pronounced in MNPs coated with oleic acid (Figure 3A) in comparison to MNP-MA and MNP-LA. By contrast, the signal intensities developed by MNP-MA were weakest with all three neutravidin concentrations. 

Regarding their nanozyme activity, the results obtained confirmed that the catalytic activity of the three MNPs significantly enhanced the colorimetric signal, thus improving visual detection by the naked eye. When comparing the measurements after the enhancement reaction with the initial ones, the enhancement effect was greater at the lowest concentration of neutravidin (0.25 mg/mL) for MNP-OA and MNP-MA, reaching a 3-fold and a 2.5-fold increase, respectively. In the case of MNP-LA, the intensity was 3.3-fold higher at the highest concentration of neutravidin (0.75 mg/mL). Concerning the possible effect of chain length, no significant differences in the enzymatic properties of the MNPs were found. Nevertheless, MNP-OA and MNP-LA showed better performance, as the signal intensities on the test line were significantly higher than those measured when using MNP-MA, before and after the enzymatic reaction. MNP-OA, despite not demonstrating the best intensity after signal enhancement, showed great signal divergences when conjugated with different concentrations of neutravidin, which could facilitate its visualization not only for qualification but also for quantification in real-case detection.

### 3.3. Effect over Time of the Nanozyme-Based Lateral Flow Assay 

**Figure 4:**
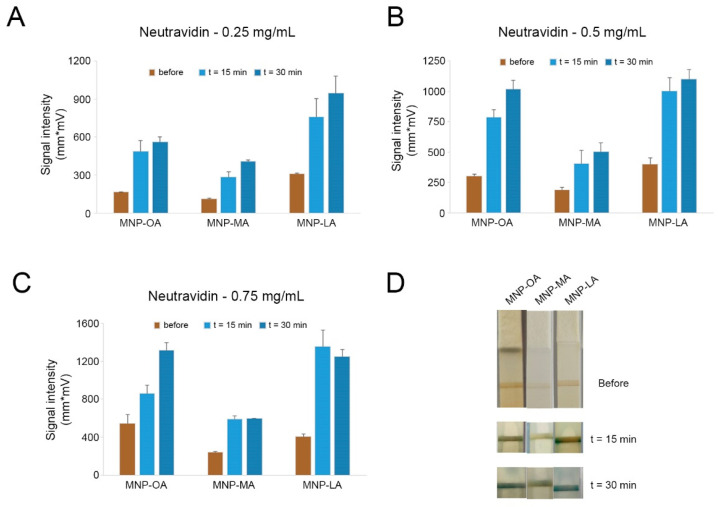
Effect over time of the peroxidase-like activity of MNP-OA, MNP-MA, and MNP-LA conjugated with three different concentrations of neutravidin: (**A**) 0.25 mg/mL, (**B**) 0.5 mg/mL, (**C**) 0.75 mg/mL. (**D**) Representative image of LFA strips at 15 min and 30 min using MNPs coated with 0.25 mg/mL of neutravidin as reporter label.

The peroxidase-like activity of the three MNPs on the strips and the effects over time were further studied. After the first signal enhancement, the strips were immersed again in the substrate solution and the reaction was left for an additional 15 min. For further comparison purposes, the signal intensities of the test lines were measured. The results for each concentration of neutravidin are shown in Figure 4. As illustrated, with neutravidin at 0.25 mg/mL and 0.50 mg/mL (Figure 4A,B), all three MNPs exhibited better peroxidase-like activities with longer reaction times in TMB and H_2_O_2_. MNP-LA still proved to be the highest caliber nanozyme in the 30 min reaction, while MNP-MA remained the lowest. Notably, the signals for MNP-OA increased significantly as the concentration of conjugated neutravidin and reaction time increased and it proved almost as capable as MNP-LA with 0.5 mg/mL neutravidin (Figure 4B), whereas when the concentration of neutravidin was 0.75 mg/mL for conjugation, the signal of MNP-MA was hardly enhanced by a longer color reaction time, which might have been due to its capability saturation as a nanozyme at high conjugate concentrations. Moreover, MNP-LA reduced from 1356.71 mm*mV in 15 min to 1249.06 mm*mV in 30 min, which might indicate instability with high levels of conjugates in catalysis. MNP-OA, however, maintained significant growth after 30 min, and this great difference could also be captured by the naked eye. These findings suggested that MNP-OA was the highest-performing option for further utilization in EV detection.

### 3.4. Nanozyme-Mediated LFIA for EV Detection

Through the experimental studies described above, MNP-OA was eventually selected as the nanozyme probe. These particles showed great performance in terms of their peroxidase-like activity in paper strips. In addition, this fatty acid coating was the first and the one most used for highly uniform and monodispersed magnetic nanoparticle synthesis in aqueous solutions [45,46]. 

**Figure 5:**
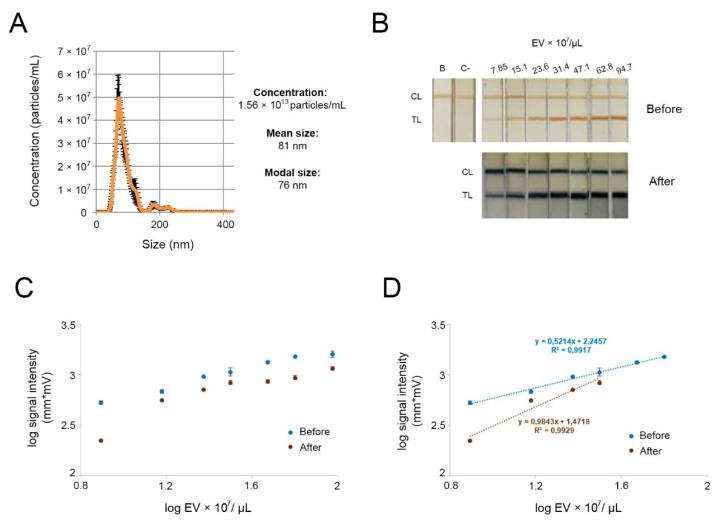
Signal enhancement for detection of different concentrations of plasma-derived EVs using anti-CD9 as capture antibody and MNP-OA-anti-CD63 as reporter label. (**A**) Concentration and hydrodynamic size distribution profiles of isolated EV, measured by NTA. (**B**) Representative image of the results obtained in the strips, before and after signal enhancement. B: blank; C-: EV-depleted plasma; CL: control line; TL: test line. (**C**) Calibration curve obtained with the LFIA optical reader, before (brown dots) and after (blue dots) signal enhancement. (**D**) Expanded view of the lower concentrations of EVs and the linear regression lines. Graphs show the means ± SDs (*n* = 3).

The nanozyme probe can target EVs and be visualized by catalyzing a color reaction with TMB and H_2_O_2_. To achieve this, the MNP-OA was conjugated with anti-CD63 antibodies, and antibodies anti-CD9 and anti-IgG were immobilized on paper biosensor membranes as test line and control line, respectively. EVs would be recognized by tetraspanin CD63 and CD9 binding to antibodies on the nanozyme probe and biosensor membrane simultaneously, forming a sandwich detecting format. This format and the use of two different tetraspanins as targets ensures that the system does not capture other non-EV components that may be present in the fractions isolated. Plasma-derived EVs obtained using a precipitation reagent were characterized by NTA to determine the concentrations of the samples and their sizes (Figure 5A). As these EVs were below 200 nm, they may be considered small EVs (sEVs), in accordance with the International Society of Extracellular Vesicles [47]. EV fractions were then diluted and a range of concentrations of the vesicles was subsequently assayed with our system. The catalysis reaction was performed in the same way as described in Section 3.3 for 30 min. The outcomes are shown in Figure 5B. A LFIA optical reader was employed to measure signal intensities before and after the catalyzation reactions. The quantification data were used to establish calibration curves (Figure 5C,D).

The test line signal intensities were significantly improved after the signal enhancement reactions with respect to both visualization and the lateral flow reader. The reproducibility of responses in the linear range before and after signal enhancement was also studied. Table 1 compares the parameters acquired from the experimental data (linear ranges, regression parameters, and LODs) of LFIA before and after signal enhancement reactions. The SD was less than 5% in all the cases, except for point 31.4 × 10^7^ EV after signal enhancement, where it was 19%. The nanozyme-mediated LFIA system showed a wider linear range (up to 62.8 × 10^7^ EV/µL) in comparison with the standard assay (up to 31.4 × 10^7^ EV/µL). The LOD was determined using the σb/m criterion, where m is the slope and σb is the y-intercept standard deviation. The LOD achieved before the enhancement is similar to that described in our previous work using MNP-OA to detect plasma-derived EVs [40]. The signal enhancement resulted in lower LOD, thus showing the potential for sensitive and decentralized analysis of biomarkers. Although this improvement is slightly better, it is within the range of interest when working with extracellular vesicles. The number of circulating EVs may be informative in itself, as shown in a pilot study with patients with chronic fatigue syndrome [30]. It is also within the ranges for further characterization (e.g., standards used for quantification of EVs by ELISA). In addition, further optimization may be applied to obtain lower limits of detection for different applications in the study of EVs, such as for the detection of other less abundant biomarkers of interest in specific diseases.

## 4. Discussion

In this paper, the peroxidase-like abilities of superparamagnetic nanoparticles with three different coatings—oleic acid, myristic acid, and lauric acid—were studied. The MNPs coated with oleic acid were ultimately used for EV detection in a lateral flow immunoassay because of their great stability and capability as nanozymes. The MNP-OAs conjugated with anti-CD63 antibodies as nanozyme probes showed three functions: recognition, visualization of EVs on the strips, and signal enhancement with their intrinsic peroxidase-like ability. Furthermore, the sensitivity and linear range of the LFIA after the signal enhancement reaction increased, while the limit of detection diminished from 5.73 x 10^7^ EVs/μL to 2.49 x 10^7^ EVs/ μL, so that the nanozyme ability of MNP-OA provided the possibility of lower-concentration detection while making it more sensitive, which is critical for high-sensitivity detection.

Since they are found in biological fluids, EVs are good candidates as non-invasive biomarkers for the diagnosis and prognosis of a variety of diseases. The composition and abundance of EVs depend on the cells of origin, as well as physiological or pathological states. The number of circulating EVs were found to be altered in several diseases [30,48,49,50] Therefore, LFIAs are useful platforms for rapid and on-site detection of circulating EVs.

Many efforts have been made recently to increase the capacity of lateral flow immunoassays. Various nanomaterials have been explored that possess various characteristics providing optical, electrical, and magnetic signals in LFIAs for biomolecular monitoring and detection. However, signal amplifications of LFIAs for naked-eye identification or quantification can be laborious and expensive. For instance, Dong et al. [51] presented EV detection with fluorescent nanospheres combined with biotinylated modification of EV membranes. Even though the method was extremely sensitive, with an LOD of 2.0 × 10^3^ EVs/μL, the sample collection and enrichment were quite time-consuming. Similarly, colloidal gold nanoparticles coupled with aptamer were applied in EV sensing by Yu et al. [52], but a long incubation time for strips of up to 1 h was not ideal for rapid detection. Other methods, such as Surface-Enhanced Raman Scattering (SERS) [53], biochips [54], and surface plasmon resonance (SPR) [55], can identify the EV contents quantitatively with valid and accurate results. However, these technologies may require the use of bulky analytical instruments or operational costs may be excessive. 

Enzyme-labeled conjugates were also proposed for LFIAs to enable signal amplification. Horseradish peroxidase (HRP) is the enzyme that has been most often used to label nanoparticles, as the oxidation of different organic substrates (such as TMB) can be catalyzed by hydrogen peroxide [56,57,58]. However, the use of natural enzymes has been limited because of their short shelf life, the possibility of inhibition or activation due to interferents in the sample matrix, denaturation at high temperatures, and acidic/alkaline pH. By comparison, nanozymes are highly robust against severe conditions and simple to manufacture by chemical synthesis, as well as having tunable catalytic activities and low costs. Many kinds of metal nanoparticles have been exploited and utilized as nanozymes in LFIAs. For example, Pt-Au NPs [59], Pt-Pb NPs [60], PB NPs [61] and MnO2-NFs [62]. They can be easily immobilized on paper-based strips and provide qualitative visual information through the collection of tracers on test lines or quantitative data by including appropriate enzymatic substrates (colorimetric or chemiluminescent detection). In addition, the magnetic features of nanozyme labels allow for rapid immunomagnetic separation. Thus, these MNPs may have potential applications in the field of enrichment of subpopulations of circulating EVs of interest. As a result, immunoassays and immunosensors that use nanozymes as signaling components have become increasingly popular in recent years, and the trend is continuing [63].

## 5. Conclusions

In conclusion, the signal-enhancement approach presented here is affordable, rapid, and does not involve the use of bulky equipment. It could also be applied for the detection of other biological substances simply by replacing the conjugated antibodies since the method is reliable and ubiquitous. Consequently, these findings imply that the nanozyme-enhanced LFIA can be exploited as a diagnostic tool for the visual assessment of biomolecules or chemical reagents and that it has potential for a variety of applications. 

## Figures and Tables

**Table 1:** Comparison of the different LODs obtained with the LFIA using magnetic nanoparticles coated with oleic acid and after signal enhancement (nanozyme-mediated LFIA).

	Linear Range	Slope (Log EVs/µL)	LOD (EVs/µL)	Regression Coefficient R^2^
**MNP-OA**	0–31.4 EVs × 10^7^/µL	0.9843	5.73 × 10^7^	0.9929
**Signal enhancement**	0–62.8 EVs × 10^7^/µL	0.5214	2.49 × 10^7^	0.9917
